# Quantitative ultrasound assessment of the influence of roughness and healing time on osseointegration phenomena

**DOI:** 10.1038/s41598-020-78806-0

**Published:** 2020-12-15

**Authors:** M. Fraulob, R. Vayron, S. Le Cann, B. Lecuelle, Y. Hériveaux, H. Albini Lomami, C. H. Flouzat Lachaniette, G. Haïat

**Affiliations:** 1 MSME, CNRS UMR 8208, Univ Paris Est Creteil, Univ Gustave Eiffel, CNRS, 61, Avenue du Général de Gaulle, 94010 Créteil Cedex, France; 2 Laboratoire d’Automatique, de Mécanique et d’informatique Industrielles et Humaines, LAMIH UMR CNRS 8201, Université Polytechnique Hauts de France, 59300 Valenciennes, France; 3 Centre de Recherche BioMédicale, Ecole Nationale Vétérinaire d’Alfort, 7 Avenue du Général de Gaulle, 94700 Maisons-Alfort, France; 4 INSERM U955, IMRB Université Paris-Est, 51 avenue du Maréchal de Lattre de Tassigny, 94000 Créteil, France; 5 Service de Chirurgie Orthopédique et Traumatologique, Hôpital Henri Mondor AP-HP, CHU Paris 12, Université Paris-Est, 51 avenue du Maréchal de Lattre de Tassigny, 94000 Créteil, France

**Keywords:** Preclinical research, Biomedical engineering, Mechanical engineering, Acoustics

## Abstract

The evolution of bone tissue quantity and quality in contact with the surface of orthopedic and dental implants is a strong determinant of the surgical outcome but remains difficult to be assessed quantitatively. The aim of this study was to investigate the performance of a quantitative ultrasound (QUS) method to measure bone-implant interface (BII) properties. A dedicated animal model considering coin-shaped titanium implants with two levels of surface roughness (smooth, *S*_*a*_ = 0.49 µm and rough, *S*_*a*_ = 3.5 µm) allowed to work with a reproducible geometry and a planar interface. The implants were inserted in rabbit femurs and tibiae for 7 or 13 weeks. The ultrasonic response of the BII was measured ex vivo, leading to the determination of the 2-D spatial variations of bone in contact with the implant surface. Histological analysis was carried out to determine the bone-implant contact (BIC) ratio. The amplitude of the echo was significantly higher after 7 weeks of healing time compared to 13 weeks, for both smooth (*p* < 0.01) and rough (*p* < 0.05) implants. A negative correlation (R = − 0.63) was obtained between the ultrasonic response and the BIC. This QUS technique is more sensitive to changes of BII morphology compared to histological analyses.

## Introduction

Endosseous cementless implants are widely employed in orthopedic, maxillofacial and dental surgery^1^. Despite a routine clinical use^2,3^, failures of implant osseointegration still occur and may have dramatic consequences leading to pain, additional surgeries and important additional costs. Implant stability is a strong determinant of the surgical success^4^.

Immediately after the surgery, the primary stability of an implant mainly depends on its design, on the surgical procedure and on the bone properties at the implantation site. Because of the damages caused during the implantation surgery, bone tissue surrounding the implant is first resorbed^5^. During the healing period lasting between a few weeks up to a few months—depending on the animal model, the implantation site and the implant’s geometry and surface roughness^6,7^—new bone is formed and remodeled leading to an osseointegrated bone-implant interface (BII)^8^. Secondary implant stability relies on the quantity and quality of bone in contact with the implant surface, ensuring the long-term stability and surgical success^8^. The implant surface roughness is known to affect primary and secondary implant stability since rough surfaces both increase the friction coefficient at the BII thus reducing micromotion just after surgery, and increase the specific surface area, thus enhancing osseointegration^9^. Assessing primary and secondary implant stability is difficult due to the complex and multiscale nature of the bone properties, their constant evolution through remodelling^10^ and the inhomogeneous implant surface roughness, which complicates the problem from a biomechanical point of view.

 Several empirical methods are used by dental and orthopaedic surgeons in order to assess primary and secondary implant stability in vivo, such as percussion tests based on the sound produced by an implant impacted by a metallic rod. However, such approaches are not reliable and depend on the surgeon proprioception. Imaging techniques such as X-ray microcomputed tomography^11^ and MRI based approaches^12^ have been suggested for the evaluation of the implant osseointegration, but their performances remain limited because of imaging artefacts due to the presence of titanium^13^ and limited image resolution, respectively.

To overcome those limitations, different biomechanical methods have been developed, in particular for dental implants. The Periotest method (Bensheim, Germany)^14^ is based on the mechanical response of the implant to an impact, monitoring the induced contact duration. However, the reproducibility of the measurements has been questioned^15,16^. The resonance frequency analysis (RFA), used in the commercialized Osstell device (Gothenburg, Sweden), records the implant first bending resonance frequency^17^, but has been related to the stiffness of the whole bone-implant structure^18^ rather than to the local properties of the BII. The RFA method thus remains limited for a direct evaluation of the biomechanical properties of the BII, independently from the larger bone environment or anatomy^19,20^.

Since ultrasonic waves are sensitive to the bone elastic properties^21^, quantitative ultrasound (QUS) techniques represent an attractive approach to evaluate primary and secondary implant stability. Our group has developed a QUS device consisting in a monoelement transducer directly screwed within a dental implant to measure its echographic response^22^. This QUS technique has been validated in vitro^22^, in silico^23–25^ and in vivo^26,27^. More recently, the results obtained for dental implants in vitro^28^ and in vivo^29^ were shown to be more reproducible and more sensitive to primary and secondary implant stability compared to data obtained using a RFA-based approach.

Despite the aforementioned promising results, it remains difficult to precisely assess the determinants of the ultrasound response of the BII due to the complex geometrical properties of clinical implants. Therefore, dedicated animal models have been developed allowing to work in a simple and standardized geometrical configuration. In particular, an animal model based on a coin-shaped titanium implant^30^ has the advantage of having a planar BII. A 200 µm thick gap^10,31^ underneath the implant surface creates a bone chamber and thus allows to clearly distinguish between mature and newly formed bone tissues. A previous feasibility study showed a dependence of the ultrasonic response on healing time for a given surface roughness and a limited number of samples^26^. However, despite these promising results, the combined effect of the surface roughness and of healing time on the evolution of the BII ultrasonic response remains unclear.

The aim of the present multimodal research study is to investigate for the first time the sensitivity of the ultrasonic response of the BII to surface roughness and healing time. Our approach consists in using a coin-shaped implant^26,30–32^, which allows to work in a standardized situation thanks to its planar interface. The influence of the implant surface roughness and of healing time on osseointegration phenomena was characterized, by comparing the results obtained using the QUS technique and histological analyses. To do so, twenty-eight coin-shaped implants having two different surface roughness levels were inserted on rabbit femurs and tibiae. Two different healing times were considered (7 or 13 weeks of implantation). After healing, the ultrasonic response of the BII was determined ex vivo and the results were compared with the bone-implant contact (BIC) ratio obtained with histological analysis. Such estimations of the BIC depending on healing time and implant surface roughness are essential to understand the osseointegration process, leading to implant secondary stability and then surgical success.

## Materials and methods

### Implant model

Thirty coin-shaped implants made of medical grade titanium Ti6Al4V alloy were prepared (diameter 5 ± 0.05 mm and thickness 3 ± 0.05 mm). The implant surface to be in contact with bone tissue was mirror polished and sandblasted with aluminium oxide (Al_2_O_3_) powders (Cobra and Basic Quattro, Renfert, Hilzingen, Germany). We chose to use sandblasting^32^ instead of for example selective laser melting (SLM) technology^33,34^ because it is a simple way to create uniform surface roughness. Two series of samples were considered with different surface roughness. The smooth (S) (respectively rough (R)) series was obtained after sandblasting the implants with 25 µm (respectively 250 µm) particles for 8 s (respectively 30 s) at 5 bar (respectively 6 bar). During sandblasting, the implant surface was maintained perpendicularly to the particle direction at a distance of 20 mm from the blasting nozzle.

**Figure 1:**
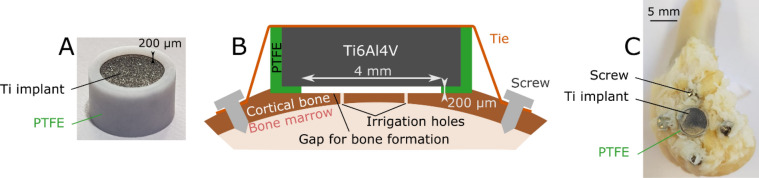
(**A**) Dedicated experimental model, designed as a coin-shaped implant, presented upside down—the bone chamber is visible (example of an implant from the R series). (**B)** Schematic cross-sectional view of the model once implanted, with the 200 µm-thick bone chamber below the implant. (**C**) Medial view of an implanted distal femur, after sacrifice.

As in^31^, all coin-shaped implants were surrounded by polytetrafluoroethylene (PTFE) caps in order to (1) limit bone growth and attachment on their lateral sides and (2) create a 200 µm-thick bone chamber between the cortical bone surface and the implant, to be filled by newly formed bone tissue during healing in order to better characterize and understand the osseointegration process (see Fig. 1A,B). During the procedure, the surgeons made sure that that all implants were inserted in cortical bone tissue.

All implants were cleaned with ethanol and put in an ultrasound bath first with absolute ethanol for 20 min and then with demineralized water for 30 min. Before surgery, they were sterilized by autoclaving (1.5 atm at 121 °C for 20 min).

### Topographical analysis

The surface profiles of one implant from both surface roughness series were analyzed using the Alicona Infinite Focus device with a × 10 objective and a resolution of 1.09 µm. The analysis was carried out first in a 3 × 3 mm region of interest located in the middle of the surface and then over 10 regions of interest (1 × 1 mm^2^) spread over the surface to assess surface heterogeneity. Each topographical analysis led to a 3D image of the surface, analyzed to extract roughness parameters such as the surface roughness *S*_*a*_, the sum of the largest peak height and pit depth *S*_*z*_, the mean dale area *S*_*da*_, the mean dale volume *S*_*dv*_ and the auto-correlation length *S*_*al*_ following a method described in^35^.

### Surgical procedure

Seven New Zealand white male rabbits (average weight 3.9 kg) were implanted with four coin-shaped implants each. Two implants were inserted medially in each posterior limb: one at the distal-medial femur and one at the proximal-medial tibia. Each left (respectively right) side received two implants from the S (respectively R) series.

After a subcutaneous injection of 0.03 mg/kg buprenorphine (Bupaq, Virbac, Carros, France) 30 min before surgery, the animals were anesthetized via intramuscular injection of 0.5 mg/kg diazepam (Valium, Roche, Basel, Switzerland), 0.25 mg/kg metedomidine hydrochloride (Domitor; Virbac, Carros, France) and 20 mg/kg ketamine hydrochloride (Ketamine1000, Virbac, Carros, France). They were intubated with a 3 mm endotracheal tube and ventilated during the whole procedure at controlled pressure with an air-oxygen mixture enriched with isoflurane (between 1.5 and 2%) in particular EtO2 > 50%.

After exposing the implantation site (medial knee), a flat bone surface of 5.6 mm diameter was levelled to (1) create a bone planar surface to receive the implant and (2) stimulate osseointegration phenomena after surgery. Four irrigation holes (Ø 0.9 mm) were drilled through the cortex to allow blood supply, and four holes (Ø 1.2 mm) were equally created around the implantation zone to stabilize the implant with osteosynthesis screws (Ø 1.6 mm, Easy Implant, Chavanod, France), attached in a cross-pattern with two elastic strings (see Fig. 1B,C).

After surgery, 25 µg/h fentanyl was transdermally delivered regularly and continuously for 3 days through a patch which could be changed once if necessary and 100 mg/L enrofloxacine (Baytril 10%, Bayer Healthcare, Loos, France) was put in water for 5 days. The animals were housed in a metal hutch (ambient temperature 19 °C and a humidity of 55%). Artificial lightening and air conditioning systems were used in the animal housing facility. The animals were fed with commercial food and water was provided ad libitum.

All animal experiments were conducted in accordance with the requirements of the European Guidelines for care and use of laboratory animals. All experimental protocols were approved by the ethical committee of the ENVA (Ecole Nationale Vétérinaire d’Alfort).

Three rabbits were euthanized after 7 weeks of implantation and the four others after 13 weeks, using an overdose of pentobarbital. The samples, consisting in the coin-shaped implants integrated in bone tissue, were carefully harvested (see Fig. 1C). Three samples were not properly osseointegrated and could not be further analyzed, while 25 implants were attached to the bone tissue.

Samples were classified based on their implant surface roughness and integration time. Four groups were created, labelled *X–Y*, where *X* represents the level of surface roughness and *Y* the number of weeks of healing time. For instance, *R-7* corresponds to the group of samples having a rough surface profile and a healing duration of 7 weeks.

### Quantitative ultrasonic device

Shortly after sacrifice, quantitative ultrasound (QUS) measurements were performed in a container filled with water at room temperature on 5 samples from group *S-7*, 6 samples from group *S-13*, 6 samples from group *R-7* and 8 samples from group *R-13*. In addition, two control implants (denoted *S-0* and *R-0*) were prepared according to the protocol described in “Implant model” section, and further analyzed topographically and using the QUS device described below, by placing them into the water tank without any contact with bone tissue. These two implants were not inserted into a rabbit and serve as references for the ultrasonic measurements.

**Figure 2:**
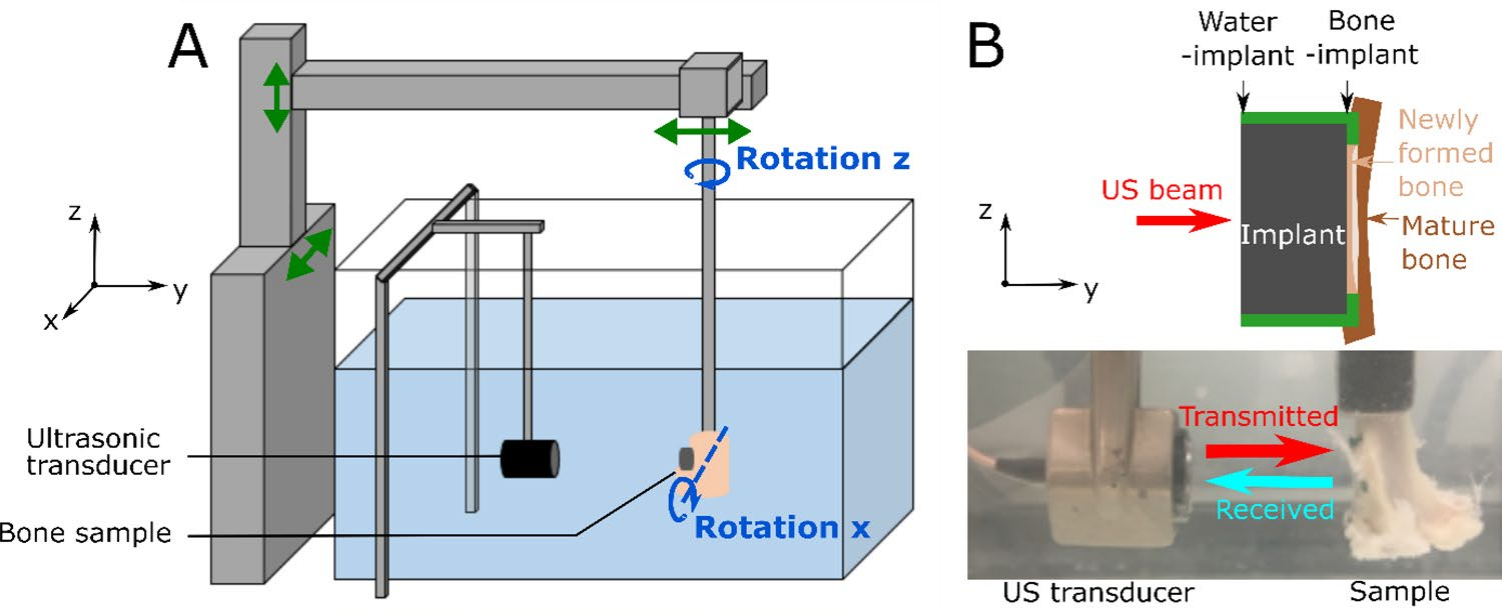
(**A**) Schematic representation of the quantitative ultrasonic device. The green arrows indicate the translation of each component. (**B**) Photo of the measurement set-up including the ultrasonic transducer acting as an emitter-receiver and the sample. The top schematic view of the implant indicates the positions of the water-implant and the bone-implant interfaces.

The QUS device shown schematically in Fig. 2A has been described in details in^26,36^. It comprised a broadband focused transducer (CMF-25; Sonaxis, Besançon, France) with a center frequency of 15 MHz, a diameter of 6 mm and a focal length of 40 mm. Assuming that the ultrasonic wave propagates into water with a velocity of *v* = 1490 m/s, this configuration leads to the beam diameter *d* at focus^37^:

$$d = \frac{\lambda F}{D} = \frac{vF}{{Df}} \cong 0.5 mm$$

However, the presence of the implant is likely to disrupt the ultrasonic beam, which leads to a defocalization of the beam and to a slight increase of the beam diameter at the focus. Note that the distance between the sample and the transducer was set by maximizing the echo of the BII, which assures that the size of the beam at the BII is minimized. The beam width at the bone-implant interface determines the lateral resolution of the measurements^37^ because it represents the region of interest where the echo of the BII is formed. The transducer worked in echographic mode and its transmitting axis was aligned in the *y*-direction (see Fig. 2). The supporting electronics included a pulse-receiver amplifier (5052A; Panametrics, Waltham, MA, USA) and an A/D conversion card of 100-MHz sampling frequency with 12-bit resolution (Spectrum, Grosshansdorf, Germany). The emitted pulse was a broadband sinusoidal impulse with a center frequency of 15 MHz and a bandwidth comprised between 7 and 20 MHz approximately.

All bone samples were carefully degassed before each measurement to remove air bubbles. The samples were hung by a clamp exposing first the water-implant interface and then the bone-implant interface to the ultrasonic beam (Fig. 2B). Both interfaces were aligned so that (1) the BII was in the *xz* plane, approximately at the focus of the transducer, and (2) the normal of the implant surface and the axis of the transducer coincided with the *y*-direction (see Fig. 2A). The parallelism between the implant and the transducer surfaces was adjusted by rotating the *x* and *z*-axes, to reach a maximum orientation error of around 1° relatively to both axes.

**Figure 3:**
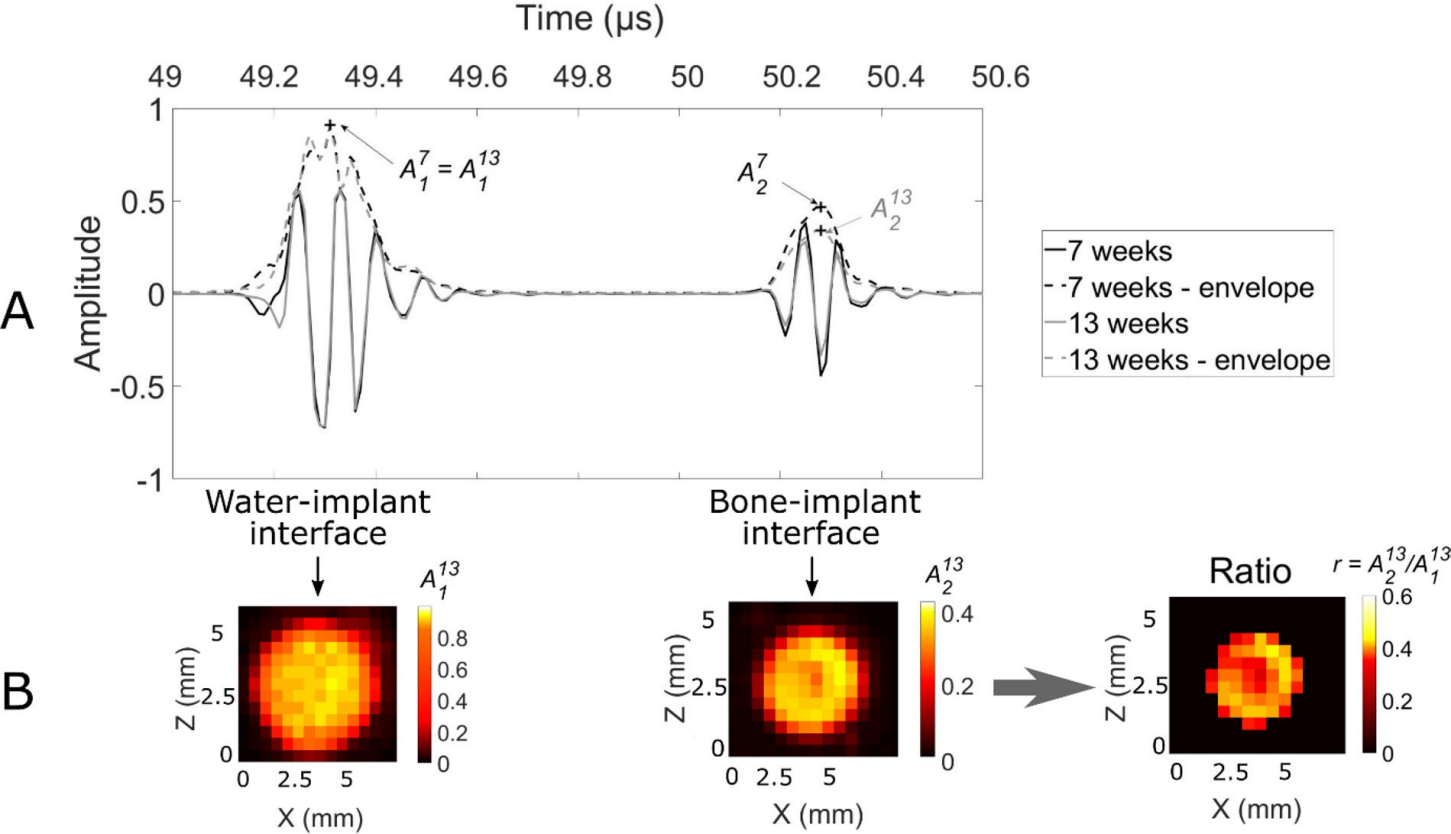
(**A**) Typical radiofrequency signals obtained for rough implants inserted for 7 (black line) and 13 weeks (grey line) and their corresponding envelopes in dashed lines. The lower script *x* in *A*_*x*_ corresponds to the interface: *1* for water-implant and *2* for implant-bone. The upper script *y* in *A*^*y*^ corresponds to the number of weeks of healing time. (**B**) 2D maps corresponding to the spatial variations of *A*_1_,* A*_2_ and *r* for a 13 week osseointegrated sample, ratios *r* = *A*_2_/ *A*_1_ are shown only for positions for which *A*_2_ > 0.21.

The displacement of the sample in the *xz* plane was controlled using two translation stages (Physik Instruments, Pantin, France), as shown in Fig. 2A. A custom-made human machine interface was developed under LabVIEW (National Instruments, Austin, TX, USA) to synchronize the displacement of the sample and the ultrasonic acquisitions. The spatial displacement step of 0.3 mm was chosen to approximately correspond to half of the ultrasonic beam diameter. 2D measurements (10 × 10 mm^2^, 256 points) were carried out for each sample (Fig. 3B) by spanning the entire coin-shaped implant surface.

### Signal processing

Figure 3A presents an example of two typical radiofrequency (*rf*) signals. For each *rf* signal (which corresponds to a given position of the sample relatively to the transducer), two echoes could be distinguished. The first echo corresponds to the water-implant interface and the second one to the BII. The envelope of each *rf* signal (dashed lines in Fig. 3A) was determined by computing the modulus of its Hilbert’s transform. The maximum amplitudes of both echoes were noted *A*_1_ (water-implant interface) and *A*_2_ (BII), and the ratio *r* = *A*_2_*/A*_1_ was computed. This normalization allowed to reduce the variability of the measurements due to (1) orientation errors of the sample relatively to the axis of the ultrasonic beam and (2) noise related errors.

Figure 3B shows typical spatial variations of *A*_1,_ *A*_2_ and *r *as a function of the sample position. For each sample *#i* (*i ∈ *{1,…,25}), a region-of-interest (ROI) was defined by considering the sample position for which *A*_2_ > 0.21, to remove *rf* signals corresponding to the periphery of the implant where the PTFE cap may have disrupted the signal. The choice of the value of 0.21 for the threshold will be discussed in the discussion. This ROI corresponded approximately to a 2D circular ROI centered on the implant, with a diameter close to 2.5 mm (non-black pixels in the ratio map in Fig. 3B).

For each sample #*i*, the average value of *r* (noted $\overline{{r_{i} }}$) as well as its standard deviation (noted $r_{i}^{sd}$) were determined by considering only sample positions within the ROI. $r_{i}^{sd}$ represents the intraspecimen variability, which corresponds to the spatial variation of $r$ among the interface illustrating the heterogeneity of bone contact at the interface. Moreover, within each sample group, the average value $\overline{r}$ and the standard deviation value $\overline{r}^{sd}$ (representing the interspecimen variability) of all $\overline{{r_{i} }}$ were calculated. The mean intraspecimen variability $r^{sd}$ was also calculated by averaging all values of $r_{i}^{sd}$ of the considered sample group.

### Histological analysis

After the QUS measurements, all samples were embedded in polymethyl methacrylate (PMMA) so that they could be cut without debonding the BII, similarly to what was done in^38^. The embedding procedure consisted in fixing the samples for 1 week in 10% phosphate-buffered formalin, rinsing them with water, dehydrating in ethanol, clearing in two baths of xylene for 12 h and finally embedding them in methyl methacrylate (MMA)^39,40^.

The embedded samples were then cut in 400 µm-thick slices with a low-speed cut-off machine (Minitom, Struers, Ballerup, Denmark) in a plane orthogonal to the implant surface. The closest slice from the center of the implant was selected and polished (LabPol-5, Struers, Ballerup, Denmark) with abrasive paper, SiC Foil with grit 1200 and 2000 using a sample holder (AccuStop, Struers, Ballerup, Denmark) to control flatness and limit the removal of material. The face to be histologically analyzed was then polished with 9-µm alumina powder and 0.3-µm alumina suspension on polishing cloths, before being colored with van Gieson picro-fuchsin and rinsed in absolute ethanol.

The stained slices, from the 5 samples of *S-7*, 6 samples of *S-13*, 6 samples of *R-7* and 8 samples of *R-13* previously studied with QUS, were analyzed by light microscopy (Stemi 305, Zeiss, Jena, Germany). The bone-implant contact (BIC), corresponding to the ratio between the length of bone in contact with the implant surface and the total length of the implant surface, was calculated using ImageJ^41^. For each sample *#i* (*i ∈ *{1,…,25}), the BIC value was determined and noted *BIC*_*i*_. For each sample group, the mean BIC value of all considered *BIC*_*i*_ was determined.

Note that we did not consider fractal analysis for investigations of the BIC^42^ because the image analysis was not precise enough. To do so, additional high-resolution imaging analyses should be performed such as micro-CT^43^ or scanning electron microscopy^42^.

### Measurements errors and statistical analyses

The measurement error of QUS, *E*_*QUS*_, corresponding to the error on the estimation of *r*, was determined for *N* = 14 samples by repeating the measurement. To do so, a first measurement was realized, leading to an average value of $r = \overline{{r_{i,1} }}$ (over the surface). Then, the sample was removed from the clamp and the measurement was repeated, leading to a second value of $r = \overline{{r_{i,2} }}$. *E*_*QUS*_ is an estimation of the reproducibility of *r* and was defined according to Eq. 1:

1 $$E_{QUS} = \frac{1}{N}\mathop \sum \limits_{i = 1}^{N} \left| {\overline{{r_{i,1} }} - \overline{{r_{i,2} }} } \right|$$

In order to assess the reproducibility of the BIC estimation using histological analysis, two consecutive slices were identically prepared and analyzed for *M* = 13 samples, leading to two values of BIC noted *BIC*_*i*,1_ and *BIC*_*i*,2_. The error corresponding to the BIC estimation *E*_*hist*_ was defined by Eq. 2:

2 $$E_{hist} = \frac{1}{M}\mathop \sum \limits_{i = 1}^{M} \left| {BIC_{i,1} - BIC_{i,2} } \right|$$

Non-parametric Mann–Whitney U-tests were performed to investigate whether the values of $\overline{{r_{i} }}$ (average ratio over a sample surface), $r_{i}^{sd}$ (intraspecimen variability or heterogeneity of the surface) and *BIC*_*i*_ were sensitive to healing time and to surface roughness. The Mann–Whitney U-test on the BIC results was performed considering all analyzed slices.

## Results

### Implant surface characterization

**Figure 4:**
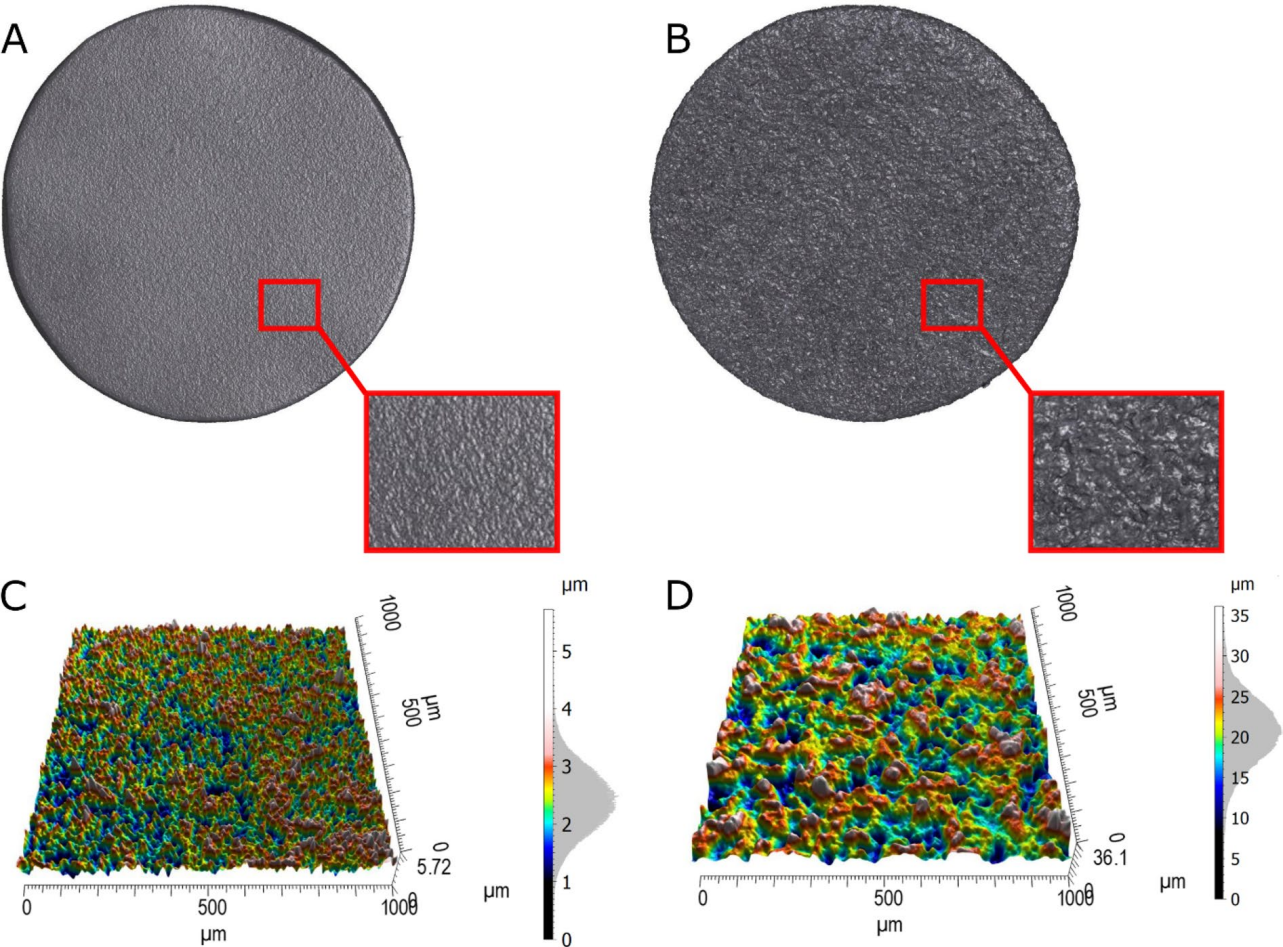
Example of implant surfaces sandblasted with (**A**) 25 µm diameter particles (S series) and (**B**) 250 µm diameter particles (R series). (**C**, **D**) Corresponding topographical results of 1 × 1 mm^2^ maps, and distribution of the measured peaks heights (Gaussian distribution on the right-handed scale).

Figure 4 shows the surface profile of two samples corresponding to the S and R series. The topographical analysis confirmed that implant of the R series presents a rougher surface with an average roughness *S*_*a*_ equal to 0.492 µm for the S series and to 3.46 µm for the R series, as indicated in Table 1.

**Table 1:** Roughness parameters (mean ± standard deviation) obtained over the entire implant surface of samples from the S and R series.

Topographic parameters	*S*_*a*_ (µm)	*S*_*z*_ (µm)	*S*_*da*_ (µm^2^)	*S*_*dv*_ (µm^3^)	*S*_*al*_ (µm)
S series	0.492 ± 0.036	6.48 ± 1.35	1371 ± 247	88.3 ± 25.9	51.7 ± 25
R series	3.46 ± 0.25	39 ± 4.61	5518 ± 620	2783 ± 582	33.9 ± 2.63

Except for *S*_*al*_, which corresponds to a distance in the plane of the surface, all other roughness parameters (*S*_*z*_,* S*_*da*_,* S*_*dv*_) are higher for the surface corresponding to implants from the R series compared to the S series.

### QUS analysis

**Figure 5:**
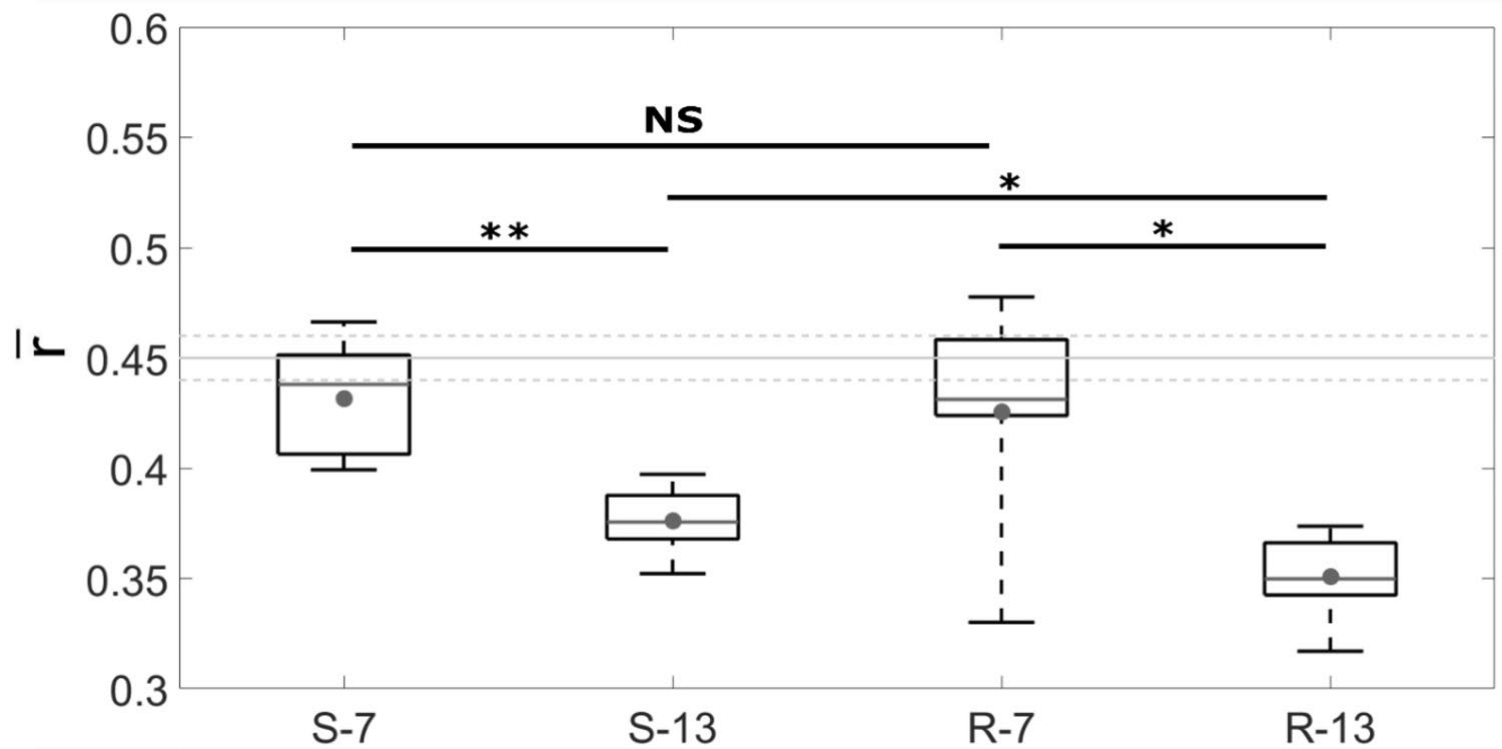
Averaged value of the ratio *r* ($\overline{r}$) for the samples belonging to each group. The dots represent the mean value, the grey lines the median value, the bottom (respectively top) edges of the box the 25th (respectively 75th) percentiles and the whiskers extend to the most extreme data points. The light grey lines correspond to the average and standard deviation for intact implants. The Mann–Whitney U-tests lead to: *p value < 0.05; **p value < 0.01; NS (non-significant difference) *p* value > 0.05.

The ultrasonic measurements are first carried out with samples from both series before surgery, which corresponds to a situation where no bone tissue is attached to the implant surface. The mean value $\overline{r}$ is equal to 0.448 ± 0.01 and 0.447 ± 0.01 for intact implants from the S and R series respectively. The corresponding results are shown with grey lines in Fig. 5.

The averaged value of the reproducibility of the QUS measurements *E*_*QUS*_ is equal to 1 × 10^–2^.

As shown in Fig. 5, for a given roughness, the average QUS ratios $\overline{r}$ are found to be significantly lower for samples with 13 weeks of healing compared to 7 weeks (*p* = 2.2 × 10^–3^ for the S series, *p* = 1.5 × 10^–2^ for the R series). No significant difference on $\overline{r}$ is obtained when considering different roughness levels after 7 weeks of healing time. However, after 13 weeks of healing time, the values of $\overline{r}$ are significantly lower for the rough implants compared to the smooth implants (*p* = 2.1 × 10^–2^).

**Figure 6:**
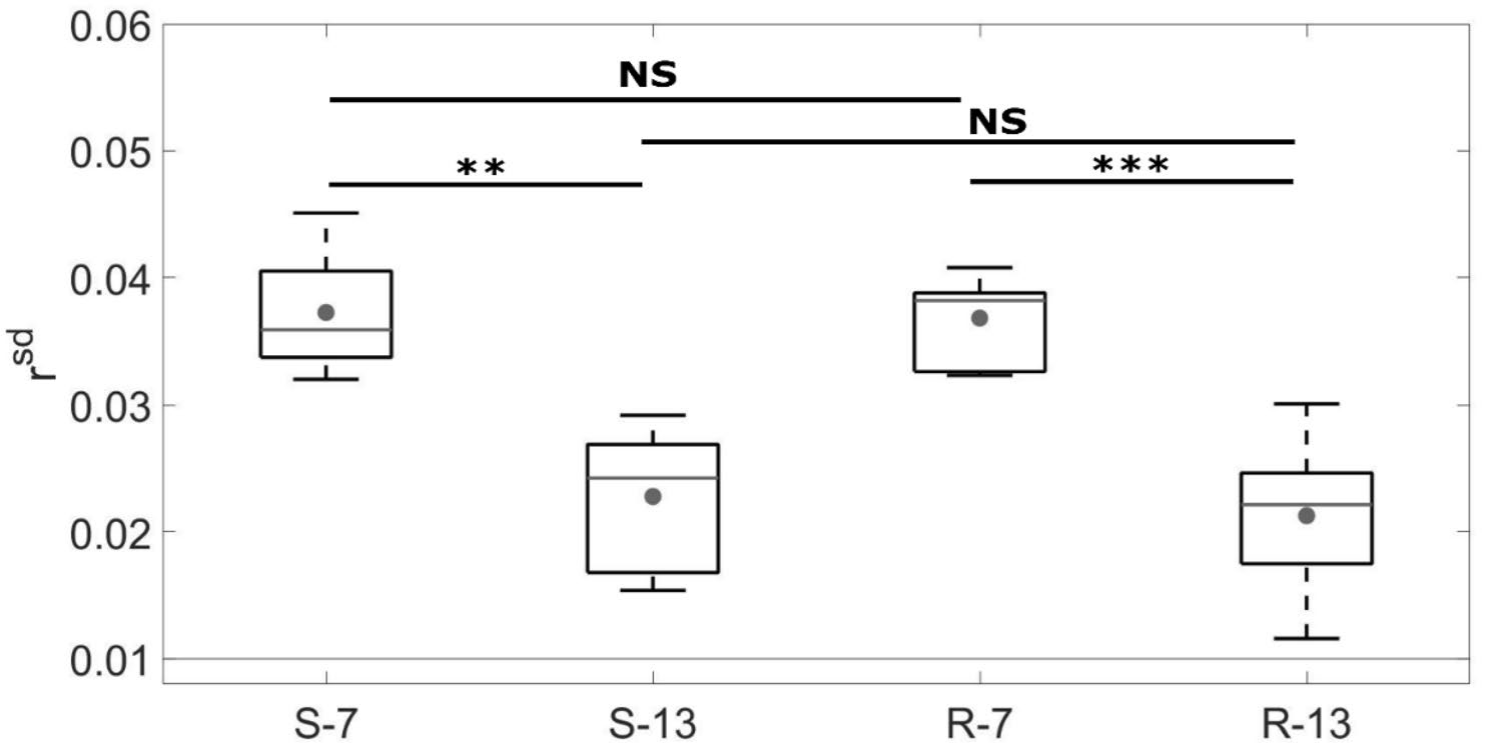
Standard deviation *r*^*sd*^ of the ratio *r* for the samples belonging to each group, which corresponds to the heterogeneity of the distribution of *r*. The dots represent the mean value, the grey lines the median value, the bottom (respectively top) edges of the box the 25th (respectively 75th) percentiles and the whiskers extend to the most extreme data points. The light grey line at 0.01 corresponds to the value for the intact implants. The Mann–Whitney U-tests lead to: **p value < 0.01; ***p value < 0.001; NS (non-significant difference) *p* value > 0.05.

Figure 6 shows the distribution of $r^{sd}$ which corresponds to the intraspecimen variability. All obtained intraspecimen variations are higher than the non-osseointegrated $r^{sd}$ (0.01, shown with grey line in Fig. 5). For each roughness level (R and S series respectively), the value of $r^{sd}$ is significantly higher after 7 weeks than after 13 weeks of healing time (*p* = 3.3.10^–4^ and 2.2.10^–3^ respectively). For a given healing time, the results are similar for the S and R series.

### Histological analysis

The reproducibility of the BIC estimation given by histological analysis *E*_*hist*_ is equal to 1.10^–1^.

**Figure 7:**
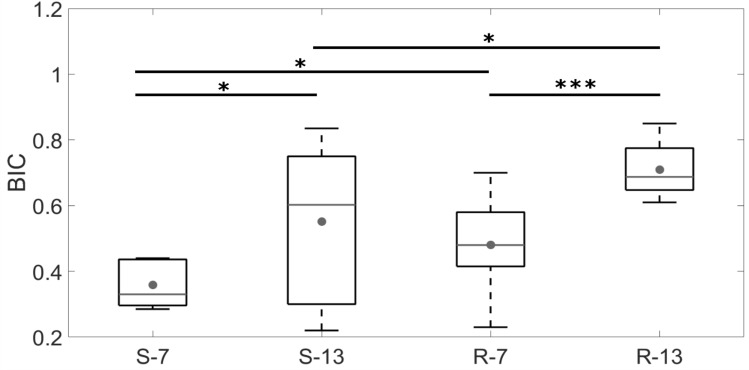
Averaged *BIC* values for the samples belonging to each group. The dots represent the mean value, the grey lines the median value, the bottom (respectively top) edges of the box the 25th (respectively 75th) percentiles and the whiskers extend to the most extreme data points. The Mann–Whitney U-tests lead to: *p value < 0.05; ***p value < 0.001.

As shown in Fig. 7, the *BIC* values are significantly higher after 13 weeks of healing time than after 7 weeks for both series of implants (*p* = 3.10^–2^ for the S series, *p* = 7.9.10^–4^ for the R series). Furthermore, for a given healing time, the averaged value of the *BIC* is significantly higher for implants of the R series compared to implants from the S series (*p* = 1.8.10^–2^ after 7 weeks, *p* = 2.5.10^–2^ after 13 weeks of healing).

### Comparison of QUS and histological measurements

**Figure 8:**
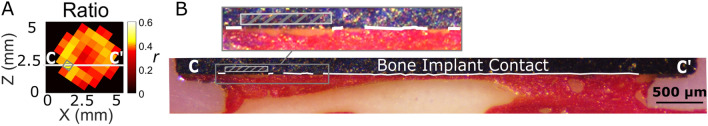
(**A**) Spatial variation of *r* obtained for a sample belonging to the group *S-13* (same as Fig. 3). (**B**) Corresponding histologic slice (in the C–C’ plane) with stained bone tissue in red. The white line corresponds to the locations where bone is in contact with the implant. The grey cross-hatched region (in **A** and **B**) corresponds to a region of interest where no bone is in contact with the implant, as observed in the histological slice (**B**) and confirmed with higher values of *r* (**A**).

Figure 8 shows a qualitative comparison for a sample belonging to the *S-13* group between the results obtained with QUS and histological analysis. The C–C’ plane in Fig. 8 represents a site-matched measurement region of interest where the grey cross-hatched area corresponds to a region of interest where no bone is in contact with the implant and where the value of *r* is the highest.

**Figure 9:**
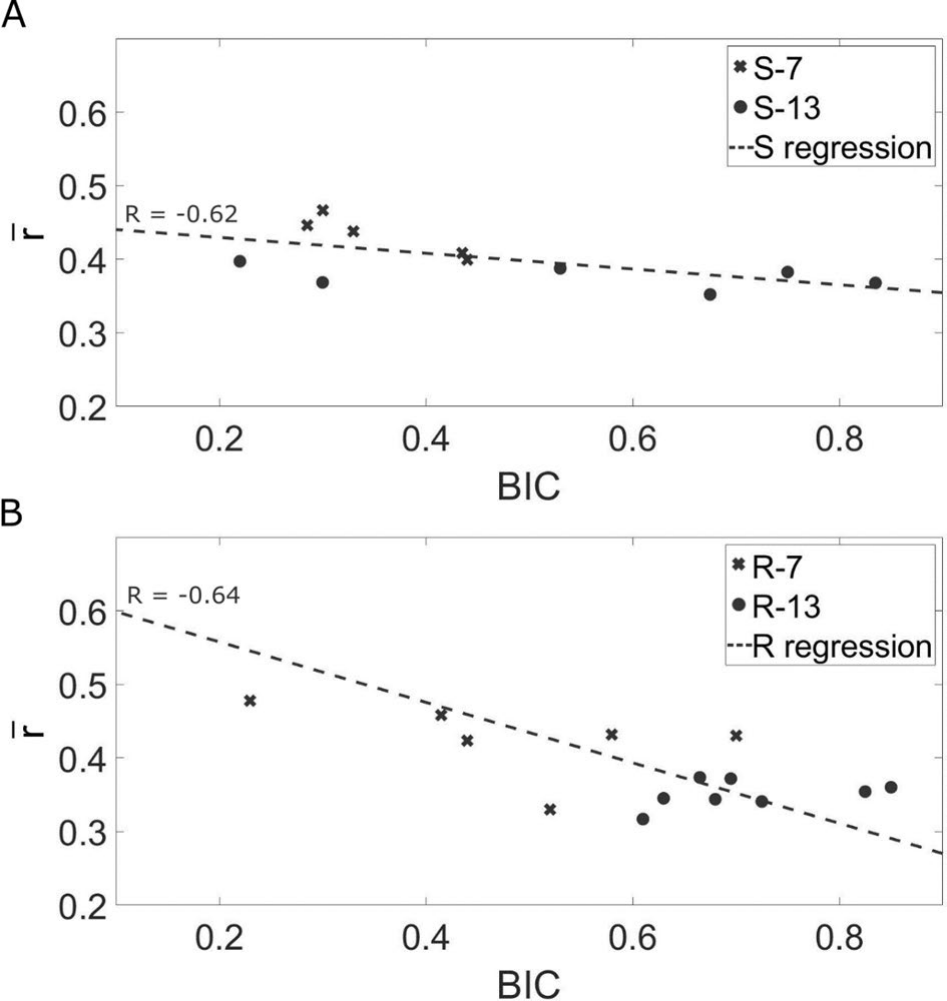
Variation of the ratio $\overline{r}$ as a function of the BIC values for all samples from the four groups of the S series (**A**) and the R series (**B**). The markers (see legend) correspond to the experimental results and the dotted lines to their linear regressions.

Figure 9 illustrates quantitatively the relationship between the QUS response of the BII and the BIC by comparing, for each sample, the values of the BIC and of $\overline{r}$ noted by a marker, which relates to the group of the sample depending on the healing time (7 or 13 weeks) and on the roughness level (S series in Fig. 9A or R series in Fig. 9B). A linear regression analysis was carried out to compare variations of $\overline{r}$ and of the BIC and led to a correlation coefficient of R = − 0.62 for the S series and of R = − 0.64 for the R series. Note that the slope of the variation of $\overline{r}$ as a function of the BIC is higher in absolute value for implants for the R series (Fig. 9B) compared to the S series (Fig. 9A), which is in agreement with Fig. 5.

## Discussion

The originality of the present study is to consider the application of a multimodal approach combining QUS and histological analyses in order to study the osseointegration process of a coin-shaped titanium implant inserted into rabbit cortical bone. To the best of our knowledge, this is the first study evidencing the effect of both surface roughness and healing time on the ultrasonic response of the BII. The animal model used in the present study is a powerful tool to investigate the interaction between an ultrasonic wave and the BII, since the planar interface allows to work under a standardized situation and to avoid multiple reflections and mode conversion arising from the complex geometries of clinical implants^25^.

The observed decrease in ultrasonic ratio $\overline{r}$ and increase in *BIC* values between 7 and 13 weeks of healing time for both roughness level is consistent with the previous QUS study on the same in vivo model showing a decrease of the average ratio from 0.53 to 0.49 and BIC increase from 0.27 to 0.69 between 7 and 13 weeks of healing time with an implant surface roughness of 1.9 µm^26^. Similar variations have been obtained using QUS method on dental implants^27,29^.

The variation of the ultrasonic response of the BII has been explained qualitatively by analyzing the evolution of the biomechanical properties of the BII and in particular of the tissue in contact with the implant surface in both experimental^26,27,29^ and numerical studies^23–25^. The reflection coefficient of an interface (which is related to *r* in the present study) increases as a function of the gap of acoustical impedance between the two materials^44^. Since the acoustical impedance of mineralized bone tissue is closer to that of titanium compared to that of non-mineralized tissue, the reflection coefficient at the bone-implant interface is affected by both (1) the amount of bone contact and (2) bone tissue properties in contact with the BII.

First, the BIC value is known to increase during bone healing, leading to more bone tissue growing in contact with the implant surface^27^. Since bone in contact with the implant surface reduces the gap of acoustical impedance, it leads to a decrease of *r*, which explains the high values of *r* obtained in the cross-hatched region of interest in Fig. 8, where no bone is in contact with the implant surface. Second, the elastic properties^38,45^ as well as mass density^45^ of newly formed bone tissue around an implant surface are known to increase as a function of healing time, which also leads to a decrease of the gap of the acoustical impedance and of the reflection coefficient. The two aforementioned phenomena have cumulative effects and can explain the negative correlation observed between $\overline{r}$ and the BIC (Fig. 9) and, more generally, the decrease of $\overline{r}$ (Fig. 5) as a function of healing time due to an increase of bone tissue quantity (Fig. 7) and quality around the implant surface. Note that other signal processing approaches such as the frequency dependence of the echo or autocorrelation analyses could be interesting to retrieve information on the BII, which are left to future studies.

The heterogeneity of the ultrasonic response over the implant surface was assessed by determining the value of the standard deviation $r_{i}^{sd}$ obtained for each sample and corresponding to the intraspecimen variability. As shown in Fig. 6, the average value *r*^*sd*^ obtained for the samples implanted in vivo is significantly higher than that for the intact samples, which may be explained by the heterogeneity of the distribution of bone tissue at the implant surface. Moreover, the average value *r*^*sd*^ obtained for the samples with 7 weeks of healing time was significantly higher than for the samples with 13 weeks of healing time, which can be explained by a higher intraspecimen variability for lower healing time. However, further studies should be carried out to understand these results due to the relatively low resolution (around 0.5 mm) of the QUS technique compared to the bone structure.

As shown in Table 1, two levels of surface roughness were considered within typical ranges corresponding to a clinical situation^30,32,46–48^. The R series presents *S*_*a*_ values around 3.46 µm, which is of the same order of magnitude as values shown to optimize osseointegration when using a comparable animal model (3.6–3.9 µm)^46^. Figure 7 shows that for both values of healing time, the BIC value is higher for samples from the R series compared to implants from the S series, which is consistent with the results obtained in^32,46^. A similar effect was obtained with QUS with a significantly higher value of $\overline{r}$ obtained for *S-13* than for *R-13* (Fig. 5). The better adhesion obtained with rougher implants is consistent the results obtained in a previous study carried out using pull-out mechanical tests^32^. However, no significant differences were observed between *S-7* and *R-7*, which can be explained by numerical results obtained by our group^49^. This numerical study shows that for a relatively low osseointegration level, the surface roughness weakly influences the value of the reflection coefficient of the BII (see in particular Fig. 4a of^49^) which depends on the properties of the material within the first 25 µm from the implant surface^49^.

Numerical simulation can also be used to explain another result obtained herein. Although the values of $\overline{r}$ obtained for implanted samples are generally lower than for intact samples, some values of $\overline{{r_{i} }} { }$ obtained after 7 weeks of healing time were found to be slightly higher than for intact samples, which seems inconsistent with the empirical description given above. Such result could actually be explained numerically by analyzing the results shown in Fig. 4a of^49^ and^50^, where a similar trend was obtained. In particular, the presence of constructive interferences (especially when a 40 µm thick soft tissue layer is present at the BII) was shown to potentially lead to higher values of the reflection coefficient compared to a fully debonded interface. Note that such behavior is more likely to be obtained for low healing time, which explains why it was not obtained for 13 weeks of healing time.

The advantage of this QUS technique is that it is a non-destructive method that can be adapted to be used in vivo^27,29^, while histological analyses are restricted to ex vivo analyses. Moreover, the QUS technique scans and determines a 2-D view of the BII while histological analyses are restricted to a 1-D view^10^ and lead to important differences according to the analyzed line (*E*_*hist*_ = 1.10^–1^).

The performances of this QUS technique can be compared to histological analysis by comparing their respective reproducibility to the interspecimen variability. While *E*_*QUS*_ is lower than the interspecimen variability $\overline{r}^{sd}$ for all four studied groups, *E*_*hist*_ is lower than the BIC standard deviations for groups *S-13* and *R-7* but not for groups *S-7* and *R-13*, which indicates that histological analysis is not sensitive enough to assess differences within samples from groups *S-7* and *R-13*.

Another way of simply comparing the performances of the QUS technique and of histological analysis is to consider the ratio *P* between the reproducibility errors (i.e., *E*_*QUS*_ and *E*_*hist*_) and the range of variation of the values obtained for the corresponding parameters (i.e., $\overline{{r_{i} }} { }$ and *BIC*_*i*_), as described in details in^51^. A low (respectively high) value of *P* indicates that the considered technique is strongly (respectively weakly) sensitive to variations of the properties of the BII. The value of *P* obtained for the QUS technique (respectively histological analysis) is equal to 0.07 (respectively 0.17), suggesting that QUS is a more sensitive method compared to histology measurements.

This study has several limitations. First, the resolution of the QUS technique is relatively low since the beam diameter is around 500 µm. Moreover, the size of the ROI where the QUS measurements were performed (diameter of around 2.5 mm) could not be increased because of the limited size of the animal model. Second, because the ultrasonic wave is first reflected from the water-implant interface before interacting with the BII, any geometrical variation or imperfection of the implant (e.g. thickness or alignment) may affect the value of *r*. The same implant geometry was considered throughout the study. The alignment of the QUS device was checked and the reproducibility of the measurements was validated for 14 samples. Third, a relatively low number of samples per group were considered, which is however of the same order of magnitude than what was done in previous studies^30,32,46–48^. The chosen sample size is a compromise between the necessary amount of data for statistical analysis and ethical considerations as well as the duration of the ex vivo experimental procedures. Moreover, it has been increased compared to the previous study using this QUS setup (only 4 animals)^26^. Fourth, the sample sandblasting was done manually, which may lead to possible surface roughness heterogeneity, which was shown in Table 1 to be around 7% of variation for the *S*_*a*_ values. Fifth, the ROI where the values of *r* were averaged was defined by comparing the value of *A*_2_ with an arbitrary threshold equal to 0.21, which corresponds to a compromise between a sufficiently high value in order to reject positions corresponding to the PTFE cap and to a sufficiently low value to obtain enough measurements in the ROI. Moreover, changing the value of the threshold within acceptable values (e.g. 0.20 to 0.22) induces slight variation of the average QUS ratio (e.g. 0.414 to 0.417) approximately three times lower than the calculated error of the technique (*E*_*QUS*_ = 0.01). Sixth, in the present study, coin-shaped implants are considered, which is a configuration different from clinical implants. However, the advantage of using such animal model is to investigate the evolution of the properties of the BII in simple and standardized conditions, which facilitate the understanding of the interaction between an ultrasonic wave and the BII. Using dental implants would complicate the interpretation of the results because of multiple reflections and mode conversion induced by their complex geometry. Such approach is necessary to understand the propagation of ultrasonic waves at the BII, to characterize the newly formed bone tissue in contact with the implant surface and to understand the osseointegration process^26^. Note that thanks to the better understanding of the interaction between an ultrasonic wave and the BII, QUS methods have been developed towards clinical application in order to evaluate the stability of dental implants using a transducer screwed into dental implants^22–25,27,29,51,52^.

## Conclusion

Quantitative ultrasound technique represents an interesting and sensitive method to investigate osseointegration phenomena, providing a 2-D mapping of periprosthetic bone quality and quantity. Combined with histological analysis, this QUS technique was shown to be sensitive to the increase of the BIC due to the increase of healing time and to changes of the implant surface roughness. The QUS technique, sensitive and non-destructive, represents a promising approach to follow osseointegration processes, which opens new paths in implantology and in particular for dental implant stability assessment^29,51^.

## Data Availability

The datasets generated and analyzed during the current study are available from the corresponding author on reasonable request.
